# A new player at the tip: KPZP steers pollen tube growth by reining in receptor trafficking

**DOI:** 10.1093/plphys/kiag226

**Published:** 2026-04-21

**Authors:** Neeta Lohani, María Flores-Tornero

**Affiliations:** Assistant Features Editor, Plant Physiology, American Society of Plant Biologists; Department of Biotechnology, Thapar Institute for Engineering and Technology, Patiala, Punjab 147004, India; Assistant Features Editor, Plant Physiology, American Society of Plant Biologists; Departament de Biologia Vegetal, Facultat de Ciències Biològiques, Universitat de València, Burjassot, Valencia 46100, Spain

Sexual plant reproduction hinges on a crucial task performed by the pollen tube, a polar and fast-growing structure that transports immotile sperm cells to the ovule, to fuse with the female gametes and complete fertilization (Johnson et al. 2019). The pollen tube grows exclusively at its tip (Steer and Steer 1989) thanks to the vesicle-mediated delivery of membrane and cell wall components to the apex (Guo and Yang 2020). While exocytosis supplies new membrane and wall material to the growing tip, endocytosis retrieves the excess membrane to prevent uncontrolled expansion. Maintaining a proper balance between exocytosis and endocytosis is essential to sustain the growth and structural integrity of the pollen tube tip during its journey to the ovule. If this delicate balance is disrupted, the tip becomes deformed and growth stalls. While many molecular players in this process have been identified, how specific regulatory proteins fine-tune the vesicle trafficking machinery at the pollen tube apex is not totally understood.

Among the key regulators of pollen tube growth in tomato are LePRK1 and LePRK2, two plasma membrane-localized receptor kinases specifically expressed in pollen tubes that sense pistil-derived and pollen-derived signals (Muschietti et al. 1998; Kim et al. 2002). Previous studies established that these receptors influence pollen tube growth through downstream effects on the actin cytoskeleton via KPP, a ROP guanine nucleotide exchange factor, and actin-binding proteins (Gui et al. 2014; Liu et al. 2020). However, whether LePRK1 and LePRK2 also influence vesicle trafficking at the tip remained an open question.

Recently in *Plant Physiology*, Pei et al. (2026) address this gap by introducing a new molecular player: Kinase Partner Zinc-finger Protein (KPZP), a cytoplasmic protein that is highly and specifically expressed in tomato pollen and pollen tubes. KPZP was identified through a yeast 2-hybrid screen as an interactor of the cytoplasmic domains of both LePRK1 and LePRK2. It belongs to the RING/U-BOX protein superfamily and contains a C-terminal RING-HC zinc finger domain. To explore its biological function, the authors generated CRISPR/Cas9 knockout mutants that, despite retaining normal fertility, showed slower pollen tube growth, establishing KPZP as a component of the rapid pollen tube growth machinery. To gain deeper insight, the authors also overexpressed KPZP, which uncovered a far more dramatic effect on pollen tube tip morphology.

Pollen tubes transiently overexpressing KPZP-eGFP in tobacco exhibited a remarkable cytoplasmic invagination at the tip, where the cytoplasm retracted inward while membranes accumulated in the resulting space between the cell wall and the invaginated cytoplasm (Fig. 1). This essentially reversed the direction of growth: instead of expanding outward, the pollen tube appeared to grow inward. Crucially, domain dissection revealed that the N terminus of KPZP, rather than its RING finger, was both necessary and sufficient to cause this invagination phenotype. Stable transgenic tomato lines overexpressing the N terminus confirmed the slower growth and tip invagination and additionally showed a higher pollen tube bursting rate. This invagination phenotype is reminiscent of what occurs when exocyst subunits such as EXO70 are overexpressed in pollen tubes (Sekereš et al. 2017), pointing to a shared disruption of tip-targeted exocytosis.

**Figure 1:**
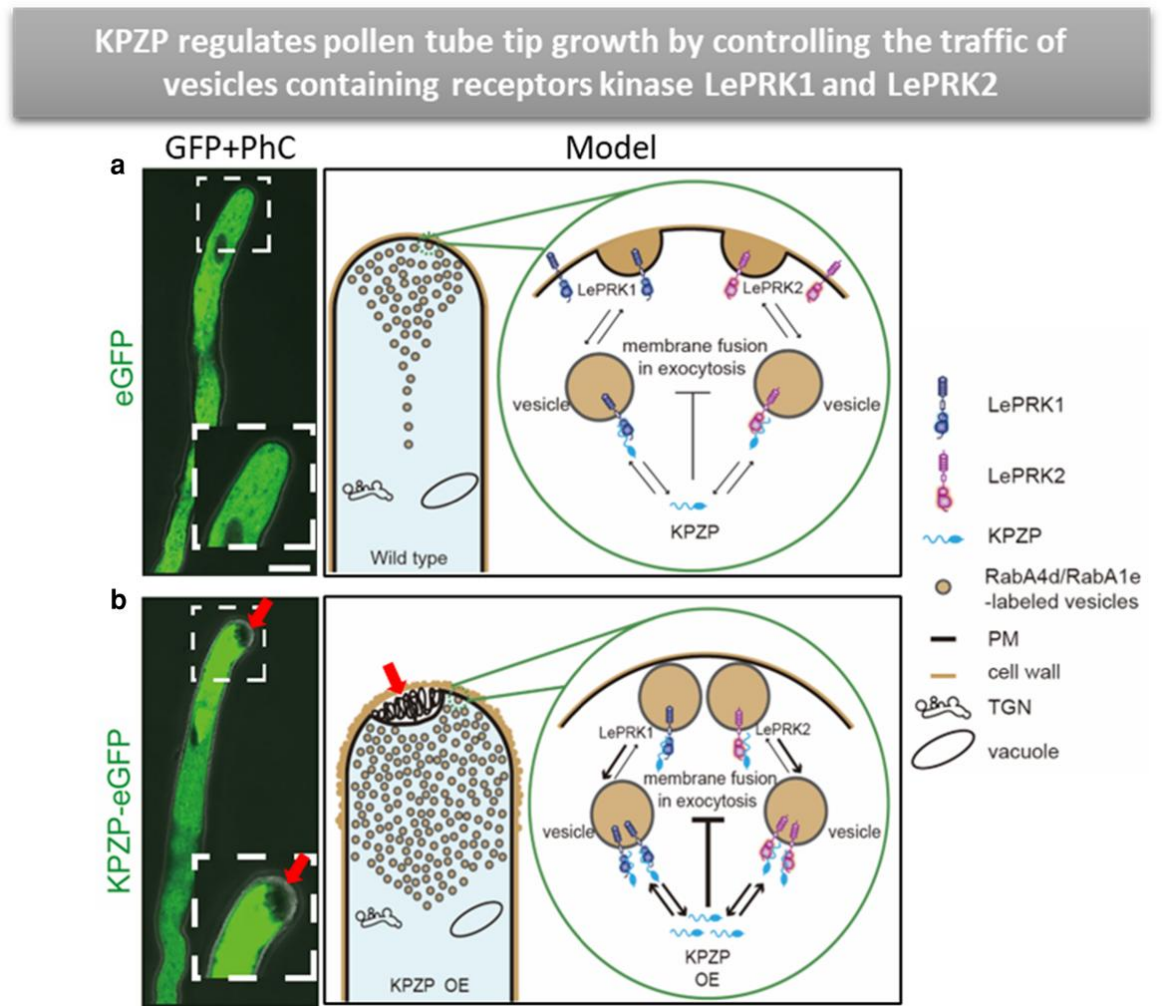
KPZP overexpression impairs vesicle fusion at the pollen tube tip, causing cytoplasmic invagination and reduced receptor delivery to the plasma membrane. a) Tobacco pollen tubes transiently expressing eGFP show normal tip growth with a well-organized inverted cone of vesicles at the apex. Under normal conditions, endogenous KPZP modulates the delivery of LePRK1- and LePRK2-containing vesicles to the plasma membrane, maintaining balanced exocytosis (right, schematic). b) Tobacco pollen tubes transiently overexpressing KPZP-eGFP display cytoplasmic invagination at the tip (red arrows), where membranes accumulate in the space between the cell wall and the retracted cytoplasm. Excess KPZP inhibits vesicle fusion with the plasma membrane, causing over-accumulation of vesicles and reduced delivery of LePRK1 and LePRK2 to the tip surface (right, schematic). Fluorescence images and schematics were adapted from Pei et al. (2026).

To unravel the underlaying mechanism of this phenotype, Pei and colleagues tracked vesicle trafficking using fluorescent markers. Both exocytic vesicles destined for the tip and recycling endosomes over-accumulated in the apical region, expanding well beyond their normal distribution zone even before visible tip deformation occurred. The distribution of phosphatidylserine, a lipid that marks vesicle fusion sites at the tip, was similarly expanded, further supporting that vesicle fusion with the plasma membrane was impaired. In contrast, FM4–64 dye uptake experiments showed that endocytosis remained largely unaffected prior to invagination, and actin organization was normal before tip deformation, ruling out both processes as the primary cause. Consistent with defective secretion, immunolabeling revealed that cell wall polysaccharides were deposited irregularly at the apex, and secretory cargo proteins carrying the signal peptides of LePRK1 or LePRK2 accumulated in the cytoplasm rather than being properly secreted.

Perhaps the most elegant finding is how KPZP functionally opposes the LePRK receptors. Previous work showed that overexpressing LePRK1 causes pollen tubes to produce outward protrusions or “blebs” at the tip (Gui et al. 2014), essentially the mirror image of the inward invagination caused by KPZP. When the authors coexpressed KPZP with LePRK1, the blebbing was suppressed and less LePRK1 reached the plasma membrane. KPZP similarly counteracted LePRK2 by redirecting it away from the plasma membrane to vacuolar membranes, canceling its growth-promoting effect. In both cases, it was the N terminus of KPZP, not its RING finger domain, that mediated the interaction. Since RING finger domains are commonly associated with ubiquitin-mediated protein degradation, this finding suggests that KPZP regulates LePRK trafficking through a distinct, nondegradative mechanism (Fig. 1).

This study adds an important new regulatory layer to our understanding of pollen tube growth. The findings by Pei et al. (2026) reveal that KPZP fine-tunes the spatial distribution of receptor kinases on the plasma membrane by modulating vesicle fusion at the growing tip, thereby ensuring precise coordination between receptor signaling and membrane trafficking. Rather than directly modulating LePRK signaling activation, KPZP controls where these receptors are deployed on the plasma membrane, defining a spatial control mechanism critical for maintaining focused polar growth. Under native conditions, KPZP may act as a buffering factor that prevents excessive receptor accumulation, stabilizing polarized growth as the pollen tube navigates changing physiological conditions. Future studies will be needed to identify additional vesicle cargo proteins that interact with KPZP and to determine how KPZP activity itself is regulated during the pollen tube's journey from the stigma to the ovule.

Recent related articles published in *Plant Physiology*:

- Fritz et al. (2025) revealed that distinct plasma membrane domains and cytoplasmic compartments at the pollen tube tip are dynamically maintained through coordinated secretory and endocytic membrane trafficking, providing a structural framework for understanding how tip growth is sustained.
- Lee et al. (2024) identified a cluster of leucine-rich repeat malectin receptor-like kinases that function in the stigma to regulate early pollen-stigma interactions, expanding the repertoire of receptor kinases involved in plant reproduction.

## Data Availability

No data is generated in this study.
